# Neurophysiological effects of transcranial alternating current stimulation combined with multidisciplinary rehabilitation in Parkinson’s disease

**DOI:** 10.3389/fpsyt.2026.1790875

**Published:** 2026-07-06

**Authors:** Tingting Hou, Keke Chen, Hongyu Zhang, Tian Zhang, Xuping Yin, Rui Huang, Haodong Sun, Zhaohui Jin, Boyan Fang

**Affiliations:** 1 Beijing Rehabilitation Medicine Academy, Capital Medical University, Beijing, China; 2 Parkinson Medical Center, Beijing Rehabilitation Hospital, Capital Medical University, Beijing, China

**Keywords:** electroencephalography, motor symptoms, neuroimaging, neuromodulation, Parkinson’s disease

## Abstract

**Introduction:**

Motor impairment in Parkinson’s disease (PD) often becomes inadequately controlled as the disease progresses. Rehabilitation therapy combined with noninvasive neuromodulation has shown benefits, yet the neural mechanisms underlying this remain unclear. This study aimed to identify neurophysiological and brain network features associated with motor improvement following high-intensity transcranial alternating current stimulation (Hi-tACS) combined with multidisciplinary intensive rehabilitation therapy (MIRT).

**Methods:**

This secondary analysis was based on a randomized controlled trial (ChiCTR2300071969). Thirty patients receiving Hi-tACS combined with MIRT were included. Responders were defined as patients achieving a minimal clinically important difference improvement greater than 3.25 points on the Unified Parkinson’s Disease Rating Scale part III. Electroencephalographic spectral analysis, resting-state functional connectivity, and dynamic brain states assessed using hidden Markov modeling were performed. Partial correlation analyses were conducted for group-differentiating measures, controlling for age and disease duration.

**Results:**

Compared with non-responders, responders showed increased temporal high beta frequencies relative power (95%CI 0.85(0.01–1.66), *P* = 0.047), reduced connectivity between the default mode and sensorimotor networks (95%CI -0.89(-1.71–0.05), *P* = 0.038), and decreased fractional occupancy of state 3 (95%CI 0.03(0.00–0.28), *P* = 0.033) and 6 (95%CI 0.02(0.00–0.35), *P* = 0.038). After Bonferroni correction, only changes in temporal high beta frequencies relative power were negatively correlated with changes in total UPDRS part III scores (R = -0.59, *P*_BON_ = 0.012) and rigidity subscores (R = -0.74, *P*_BON_ < 0.001).

**Discussion:**

These results suggest that motor improvement following combined Hi-tACS and MIRT in PD patients is associated with alterations in brain oscillations and network connectivity, particularly in high-beta frequencies. The findings contribute to understanding the neural mechanisms underlying rehabilitation and neuromodulation in PD. Future studies should investigate the long-term effects of this intervention and its applicability in larger, more diverse populations. Moreover, exploring additional biomarkers may help identify individuals who are most likely to respond to this treatment.

**Clinical Trial Registration:**

https://www.chictr.org.cn, identifer ChiCTR2300071969.

## 1 Introduction

Parkinson’s disease (PD) is a progressive neurodegenerative disorder in which motor impairments, including bradykinesia, rigidity, tremor, and postural instability, represent the principal contributors to disability and reduced quality of life (1). Pharmacological treatment based on dopamine replacement therapy remains the foundation of PD management and provides substantial symptomatic benefit in the early stages of the disease. However, with disease progression, the long-term effectiveness of medication is frequently compromised by motor fluctuations and dyskinesias, while axial symptoms and rigidity often respond insufficiently to pharmacological intervention (2). These limitations highlight the need to explore adjunctive therapeutic strategies beyond medication that may improve motor function through alternative neurobiological mechanisms.

In this context, multidisciplinary intensive rehabilitation therapy (MIRT) has attracted increasing attention and has been shown to improve motor performance, balance, and gait function in patients with PD (PwP) (3). By integrating task-oriented training, aerobic exercise, and sensorimotor stimulation, MIRT aims to facilitate motor recovery through experience-dependent neural plasticity (4). Our previous research has demonstrated that MIRT alone can enhance motor function in PD by promoting a transition from sensory processing to higher-order cognitive control within neural networks (4). However, despite its benefits, MIRT alone does not always lead to consistent or significant improvements across all patients, especially in those with advanced stages of the disease or those with limited neuroplasticity due to aging. At the same time, noninvasive brain stimulation techniques such as transcranial alternating current stimulation (tACS) have emerged as neuromodulatory approaches capable of externally influencing neural oscillatory activity and large-scale brain network interactions (5, 6). In particular, high-intensity tACS (Hi-tACS) differs from conventional low-intensity stimulation in that it produces widespread effects at the whole-brain level and is capable of modulating activity in deeper brain regions (7). Therapeutic benefits and safety of Hi-tACS have been reported in conditions including major depressive disorder, chronic insomnia, and adult attention deficit hyperactivity disorder (8–11). Importantly, the combination of neuromodulation and rehabilitation training may result in synergistic effects, whereby stimulation facilitates the engagement of relevant neural circuits and enhances the efficacy of subsequent behavioral training (12). This combination is particularly advantageous for PwP as it may overcome the limitations of rehabilitation alone and provide more robust motor recovery through enhanced cortical and subcortical network engagement. Despite these potential advantages, our parent randomized controlled trial (RCT) recently revealed that although the addition of Hi-tACS to MIRT resulted in motor improvements, the group-level difference between the active and sham interventions was not statistically significant (13). This lack of a group-level disparity underscores a profound inter-individual variability in clinical response, making it imperative to investigate the neural mechanisms that drive treatment success in specific individuals within the combined intervention framework.

A comprehensive understanding of neural mechanisms underlying motor improvement in PD requires a multilevel analytical framework that captures complementary aspects of brain function. At the electrophysiological level, abnormal neural oscillations, especially within the beta frequency range, have been closely associated with motor impairment (14, 15), although their functional significance may vary across cortical regions and specific frequency sub-bands (16). Recent studies have also highlighted that neuroplastic changes induced by noninvasive brain stimulation techniques, such as tACS, can modulate these oscillatory patterns, potentially restoring normal motor function in PwP (6, 17). This modulation is particularly important for improving cortical and subcortical interactions, which are often disrupted in PD. At the network level, functional magnetic resonance imaging (fMRI) studies have demonstrated disrupted interactions among the sensorimotor network (SMN), default mode network (DMN), and cognitive control network (CCN), suggesting that motor dysfunction in PD may arise from large-scale network reorganization rather than isolated regional abnormalities (18). However, static measures of oscillatory power and functional connectivity (FC) are limited in their ability to reflect the temporal dynamics of brain activity. Hidden Markov models (HMM) represent a data-driven approach that enables characterization of transient whole-brain connectivity states and their temporal occupancy, thereby providing insight into the efficiency of transitions between distinct functional configurations (19). Recent advances in dynamic brain network research, particularly using HMM, have shown that PD is characterized by inefficient transitions between brain states, which may contribute to impaired motor control (20). Together, electrophysiological, network-based, and dynamic analyses offer complementary perspectives for elucidating treatment-related neural mechanisms.

Based on these considerations, the present study aimed to investigate the neural regulation and brain network mechanisms associated with motor improvement induced by combined Hi-tACS and MIRT in PwP. Participants were further classified into responder and non-responder groups according to the minimal clinically important difference (MCID) of the Unified Parkinson’s Disease Rating Scale, Part III (UP-DRS-III) (21). By integrating electroencephalographic spectral analysis, resting-state FC assessed using fMRI, and dynamic network analysis based on HMM, this study aimed to identify neurophysiological and brain network features associated with inter-individual variability in motor improvement, and to explore how oscillatory modulation, large-scale network interactions, and dynamic brain state organization relate to treatment responsiveness.

## 2 Materials and methods

### 2.1 Study design and ethical approval

This secondary analysis was based on a randomized, double-blind, sham-controlled clinical trial investigating the effects of Hi-tACS combined with MIRT in PwP. The original trial was registered with the Chinese Clinical Trial Registry (ChiCTR2300071969), and its methodology, intervention protocols, outcome measures, and assessment schedule have been published previously as a study protocol (22). To specifically investigate the neurobiological basis underlying the responsiveness to the combined treatment, this secondary analysis *a priori* excluded the sham-stimulation group from the parent trial and exclusively enrolled patients who received the active Hi-tACS combined with MIRT intervention.

The trial was conducted at Beijing Rehabilitation Hospital, Capital Medical University (Beijing, China). Ethical approval was obtained from the Ethics Committee of Beijing Rehabilitation Hospital, Capital Medical University (Ethical Review No. 2023kky064). All participants provided written informed consent before enrollment, and all procedures were conducted in accordance with the Declaration of Helsinki.

### 2.2 Participants and group stratification

**Figure 1:**
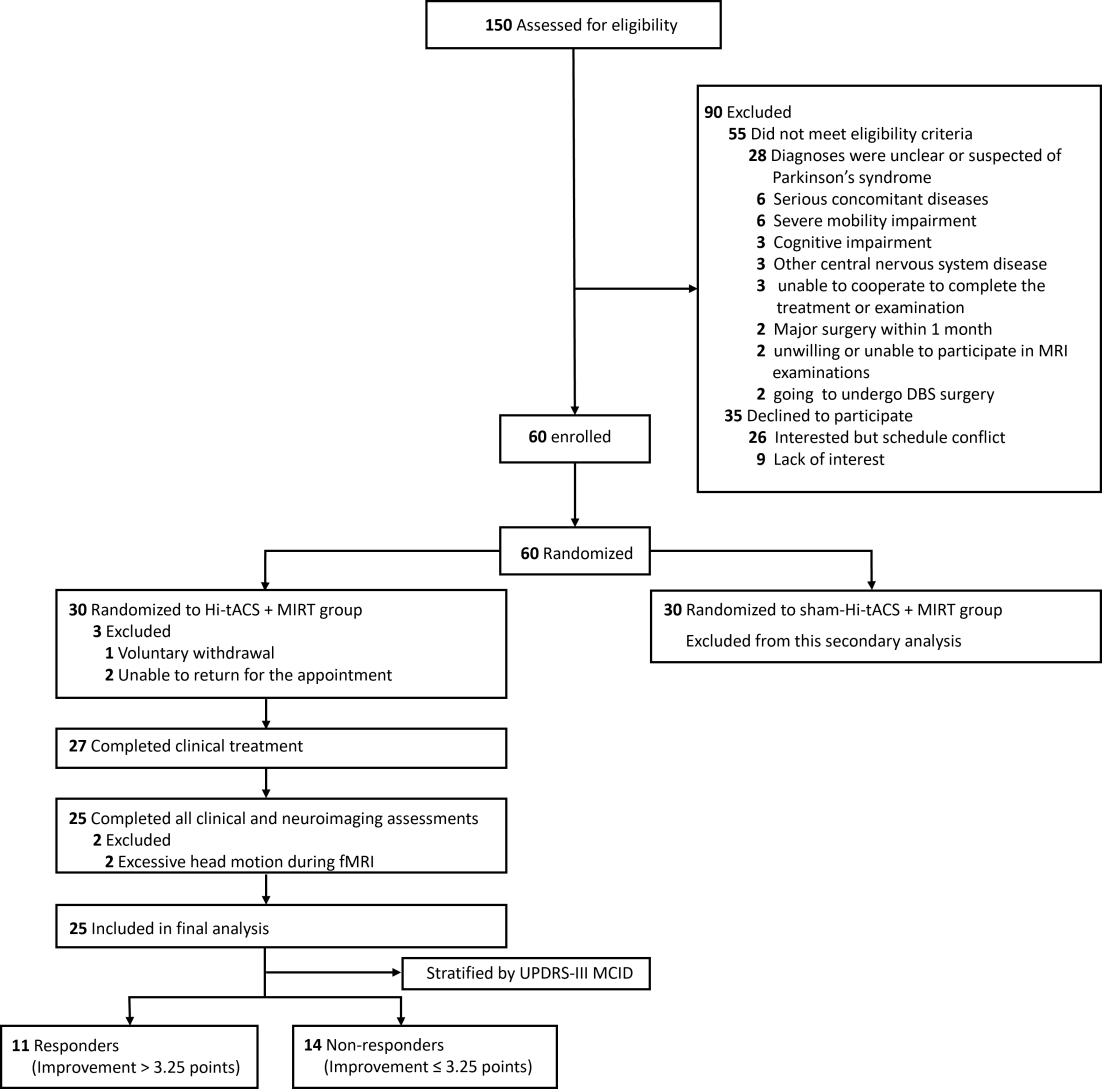
CONSORT diagram of study flow.

Between October 2023 and January 2025, the parent clinical trial recruited individuals aged 45 to 70 years diagnosed with idiopathic PD (Hoehn and Yahr stages 1–3). To ensure cohort homogeneity, eligible patients were required to exhibit motor deficits necessitating rehabilitation, stable dopaminergic medication usage, independent ambulation, and a minimum of primary education. Patients were strictly excluded if they presented with atypical or secondary Parkinsonism, prior deep brain stimulation, epilepsy, MRI contraindications, or concurrent neurological disorders. All enrollees provided written informed consent. While the parent trial employed a rigorous double-blind, randomized design comparing active Hi-tACS versus sham stimulation, the present secondary analysis exclusively focused on the 30 patients allocated to the active Hi-tACS + MIRT arm. All participants were drawn from an RCT and underwent the same active intervention protocol. Motor response was evaluated using the UPDRS-III scores. Treatment-related motor improvement was defined as the change in UPDRS-III scores between baseline and immediately after the intervention. Based on established criteria for the MCID (21), patients exhibiting an improvement greater than 3.25 points were classified as responders. Patients who did not reach this threshold were classified as non-responders (**Figure 1**).

### 2.3 Intervention protocol

Over a consecutive 10-day period, patients received a total of 20 active Hi-tACS sessions (two 40-minute sessions per day, separated by at least 6 hours). The stimulation was delivered using FDA-cleared Nexalin devices, which generated a 77.5 Hz square-wave current with an amplitude of 15 mA (zero-to-peak). For electrode positioning, a primary rectangular pad (4.45 × 9.53 cm) was applied across the frontal region spanning Fpz, Fp1, and Fp2, while two smaller pads (3.18 × 3.81 cm) were placed over the bilateral mastoids. To ensure patient comfort and safety, each 40-minute stimulation block incorporated a 3-minute (180-second) gradual ramp-up at the beginning and a 12-second ramp-down phase at the end.

Alongside the 10-day neuromodulation period, all patients engaged in an intensive, multidisciplinary rehabilitation regimen consisting of four specific daily training components. This standardized MIRT program featured: ① 40 minutes of group-based physical therapy (four patients per group) emphasizing warm-ups, flexibility, and stretching exercises; ② 30 minutes of personalized, technology-assisted gait and balance training utilizing the Balance Tutor (Meditouch, Israel) and C-MiLL (Motek, Netherlands) platforms; ③ 30 minutes of aerobic conditioning on a NuStep T5XR cross-trainer; and ④ an hour-long group speech therapy session. To maintain treatment uniformity and eliminate operator-related bias, all training modules were consistently supervised by the same team of certified rehabilitation specialists.

### 2.4 Clinical outcome assessment

Motor function was assessed using the UPDRS-III scores, which were administered in the medication-off state, defined as at least 12 hours after withdrawal of dopaminergic medication. For the purposes of this secondary analysis, motor outcomes assessed at baseline (T0) and immediately after the ten-day intervention (T1) were included, as later in-person motor assessments were substantially affected by follow-up constraints. At both T0 and T1, resting-state electroencephalography (EEG) with eyes closed and resting-state fMRI (rs-fMRI) were acquired, with neurophysiological and neuroimaging assessments conducted in close temporal proximity to the clinical motor evaluations.

### 2.5 EEG acquisition and spectral analysis

The EEG was acquired using a 64-channel EEG cap (model wave-guard™ original 64, Netherlands) to enable real-time recording of the EEG signal (23). Electrodes were arranged in accordance with the worldwide 10/20 system, utilizing bilateral ear mastoids as reference electrodes. The sampling frequency was established at 1000 Hz, and band-pass filtering was applied between 1 and 80 Hz. Furthermore, measures were implemented to keep electrode impedance under 10kΩ to reduce interferences during data acquisition (24). The collected data was stored on a computer for offline processing. Relative power in the theta (4–8 Hz), alpha (8–13 Hz), low-beta (14–19 Hz), high-beta (20–30 Hz), low-gamma (31–45 Hz), and high-gamma (46–80 Hz) frequency bands were calculated as the ratio of band-specific power to the total power across all frequencies.

### 2.6 MRI acquisition, functional connectivity and hidden markov model analysis

fMRI protocol Structural 3D T1-weighted and rs-fMRI data were collected using a 3.0t MRI scanner (GE SIGNA Pioneer) (25, 26). The imaging dataset included T1-weighted, T2-weighted, fluid-attenuated inversion recovery, and T2-star weighted angiography magnetic resonance images. A total of 240 volumes of rs-fMRI data were acquired. Participants were instructed to close their eyes and achieve a state of relaxation while maintaining wakefulness. Preprocessing included DICOM-to-NIfTI conversion, removal of initial 10 volumes, slice timing correction, motion realignment (Fris-ton 24 parameters), T1 co-registration via unified segmentation (SPM12, http://www.fil.ion.ucl.ac.uk/spm), linear detrending, nuisance regression (Friston 24 motion, white matter, and CSF signals), and bandpass filtering.

rs-fMRI time series were extracted from 160 regions of interest defined by the Dosenbach atlas. Regions corresponding to the cerebellum (ROIs 143–160) were excluded to minimize potential confounding effects, leaving 142 cortical regions for subsequent analyses. These regions were assigned to five large-scale functional networks, including the default mode, fronto-parietal, cingulo-opercular, sensorimotor, and occipital networks, which were used for FC analyses.

HMM was utilized to delineate the dynamic characteristics of extensive brain networks derived from rs-fMRI time series. The investigation utilized the HMM-MAR toolkit in MATLAB, where each hidden state was represented as a multivariate Gaussian distribution. Each state was characterized by a state-specific mean vector and covariance matrix, representing the average functional activity (AFA) and FC patterns associated with that state, respectively (27). The covariance matrix quantified the level of functional coupling among brain areas in a specific state, whereas the mean vector indicated the average BOLD signal amplitude across regions. In accordance with prior research, the quantity of concealed states was methodically adjusted from 2 to 20 (28, 29). For each candidate model, the variational free energy was calculated as an indicator of model evidence, and five repetitions were conducted for each state number to guarantee model stability. The configuration with the minimal free energy was chosen as the best state (30). State-specific FC matrices and mean BOLD time series, designated as AFA, were derived utilizing the covariance (cov) and mean (mu) functions from the HMM-MAR toolkit. In addition to state-wise spatial patterns, several temporal metrics were derived to characterize brain state dynamics, including fractional occupancy (FO), defined as the proportion of time spent in each state; switching rate (SR), reflecting the frequency of transitions between states and indexing the stability of brain dynamics; and mean dwell time (MDT), defined as the average number of consecutive time points spent in a given state before transitioning to another state (28).

### 2.7 Statistical analysis

Statistical analyses were performed using SPSS version 27.0. Data normality was assessed using the Shapiro–Wilk test. For all clinical, EEG, and fMRI measures included in the present secondary analysis, treatment-related changes were calculated as the difference between post-intervention (T1) and baseline (T0) values (Δ = T1 − T0). These change scores were used for all subsequent group comparisons and correlation analyses.

Continuous variables are presented as mean ± standard deviation or median (inter-quartile range), as appropriate, and categorical variables as counts and percentages. Between-group differences in change scores were assessed using independent samples t-tests or Mann–Whitney U tests, depending on data distribution.

Associations between neurophysiological measures and motor outcomes were examined using partial correlation analyses based on change scores (ΔT1 − T0), controling for age and disease duration. To account for multiple comparisons, the Bonferroni correction was applied. A two-tailed *P* value < 0.05 was considered statistically significant.

## 3 Results

### 3.1 Study population and baseline characteristics

A total of 30 patients receiving Hi-tACS combined with MIRT were initially included. During the intervention period, three patients withdrew from the study: one withdrew voluntarily, and two were unable to attend scheduled treatment sessions. Of the remaining 27 patients, rs-fMRI data from two participants were excluded due to excessive head motion. Consequently, 25 patients with complete clinical, EEG, and fMRI data were included in the final analysis.

Based on the MCID of the UPDRS-III scores, patients with an improvement greater than 3.25 points were classified as responders (n = 11), while those who did not reach this threshold were classified as non-responders (n = 14). Baseline demographics were well-balanced between groups (**Table 1**).

**Table 1:** Demographics and clinical data in both groups before and after Hi-tACS combined with MIRT.

Characteristics	Responding group (n = 11)			Nonresponding group (n = 14)			Baseline
	Before mean (SD)	After mean (SD)	P value	Before mean (SD)	After mean (SD)	P value	P value
Age, median, y	65.36(4.08)			61.93(6.90)			0.16^c^
Sex, n (%)							0.53^b^
Male	4(36.4)			6(42.9)			
Female	7(63.6)			8(57.1)			
Affected side, n (%)							0.43^b^
Left	6(54.5)			6(42.9)			
Right	5(45.5)			8(57.1)			
Hoehn & Yahr (off^a^), n (%)							0.85^b^
1.0	0(0.0)			0(0.0)			
1.5	2(18.2)			4(28.6)			
2.0	6(54.5)			8(57.1)			
2.5	2(18.2)			1(7.1)			
3.0	1(9.1)			1(7.1)			
Disease Duration, y	6.91(2.66)			6.64(2.56)			0.80^c^
Treatment duration, y	5.82(3.06)			5.29(2.20)			0.62^c^
Years of education, y	12.64(2.69)			12.64(3.48)			0.99^c^
LEDD	584.59(177.73)			528.36(209.71)			0.49^c^
UPDRS III score	28.18(11.11)	17.45(5.37)	0.001^e^	22.29(9.04)	23.93(8.54)	0.058^e^	0.16^c^
UPDRS III subscale score							
Tremor	4.82(5.60)	3.36(3.72)	0.091f	3.50(4.11)	3.36(4.31)	0.632^f^	0.94^d^
Bradykinesia	12.55(4.97)	6.73(2.41)	0.003f	10.07(6.07)	11.71(5.18)	0.099^f^	0.13^d^
Rigidity	5.27(2.87)	3.09(2.77)	<0.001^e^	4.50(3.11)	4.29(2.97)	0.701^e^	0.53^c^
Postural	3.36(2.34)	2.27(1.49)	0.024^f^	2.29(1.33)	2.50(1.61)	0.595^f^	0.24^d^

Hi-tACS, high-intensity transcranial alternating current stimulation; MIRT, multidisciplinary in-tensive rehabilitation therapy; SD, standard deviation; UPDRS III, Unified Parkinson’s Disease Rating Scale part III;.

^a^ ”Off” standed for “off state” of levodopa treatment (≥12 hours post-levodopa).

^b^ Chi-squared test.

^c^ Independent-samples test.

^d^ Mann–Whitney U test.

^e^ Paired-sample test.

^f^ Wilcoxon signed-ranks test.

### 3.2 EEG frequency-domain analysis

**Figure 2:**
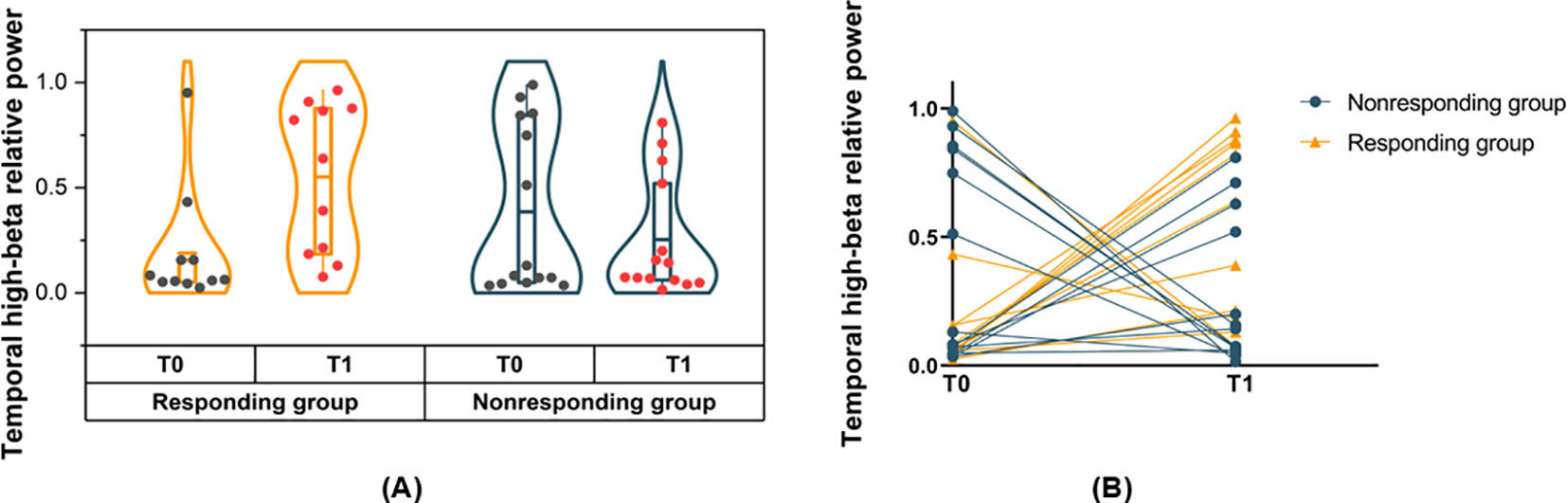
Temporal high-beta relative power before and after the intervention. **(A)** Violin plots displaying the distribution of temporal high-beta relative power at baseline (T0) and post-intervention (T1) for both the responding and nonresponding groups. The internal boxplots represent the mean values, with overlaid scatter points showing individual patient values. **(B)** Spaghetti plots illustrating the individual longitudinal trajectories of temporal high-beta relative power from T0 to T1. Each line represents a single participant, with dark blue circles denoting the nonresponding group and orange triangles denoting the responding group. (T0, baseline; T1, post-intervention).

A significant between-group difference was found in the pre–post intervention change of temporal high-beta (20–30 Hz) relative power (95%CI 0.85(0.01–1.66), *P* = 0.047), with responders showing an increase and non-responders showing a decrease (**Figure 2**). No other frequencies’ relative power demonstrated statistically significant between-group differences.

### 3.3 Functional connectivity analysis

**Figure 3:**
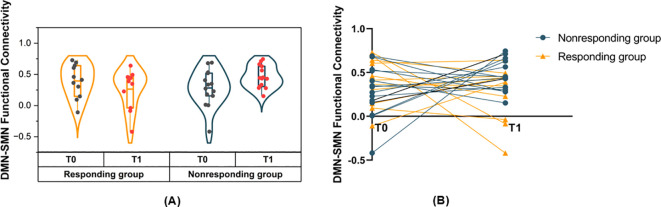
DMN–SMN functional connectivity before and after the intervention. **(A)** Violin plots displaying the distribution of DMN-SMN functional connectivity at baseline (T0) and post-intervention (T1) for both the responding and nonresponding groups. The internal boxplots represent the mean values, with overlaid scatter points showing individual patient values. **(B)** Spaghetti plots illustrating the individual longitudinal trajectories of DMN-SMN functional connectivity from T0 to T1. Each line represents a single participant, with dark blue circles denoting the nonresponding group and orange triangles denoting the responding group. (DMN, default mode network; SMN, sensorimotor network; T0, baseline; T1, post-intervention).

In the rs-fMRI FC analysis, a significant between-group difference was observed in the connectivity strength between the DMN and the SMN (95%CI -0.89(-1.71–0.05), *P* = 0.038). Compared with responders, non-responders exhibited an increase in DMN–SMN FC, whereas responders showed a relative decrease in connectivity strength between these two networks (**Figure 3**). No other network FC demonstrated statistically significant between-group differences.

### 3.4 Hidden markov model analysis

**Figure 4:**
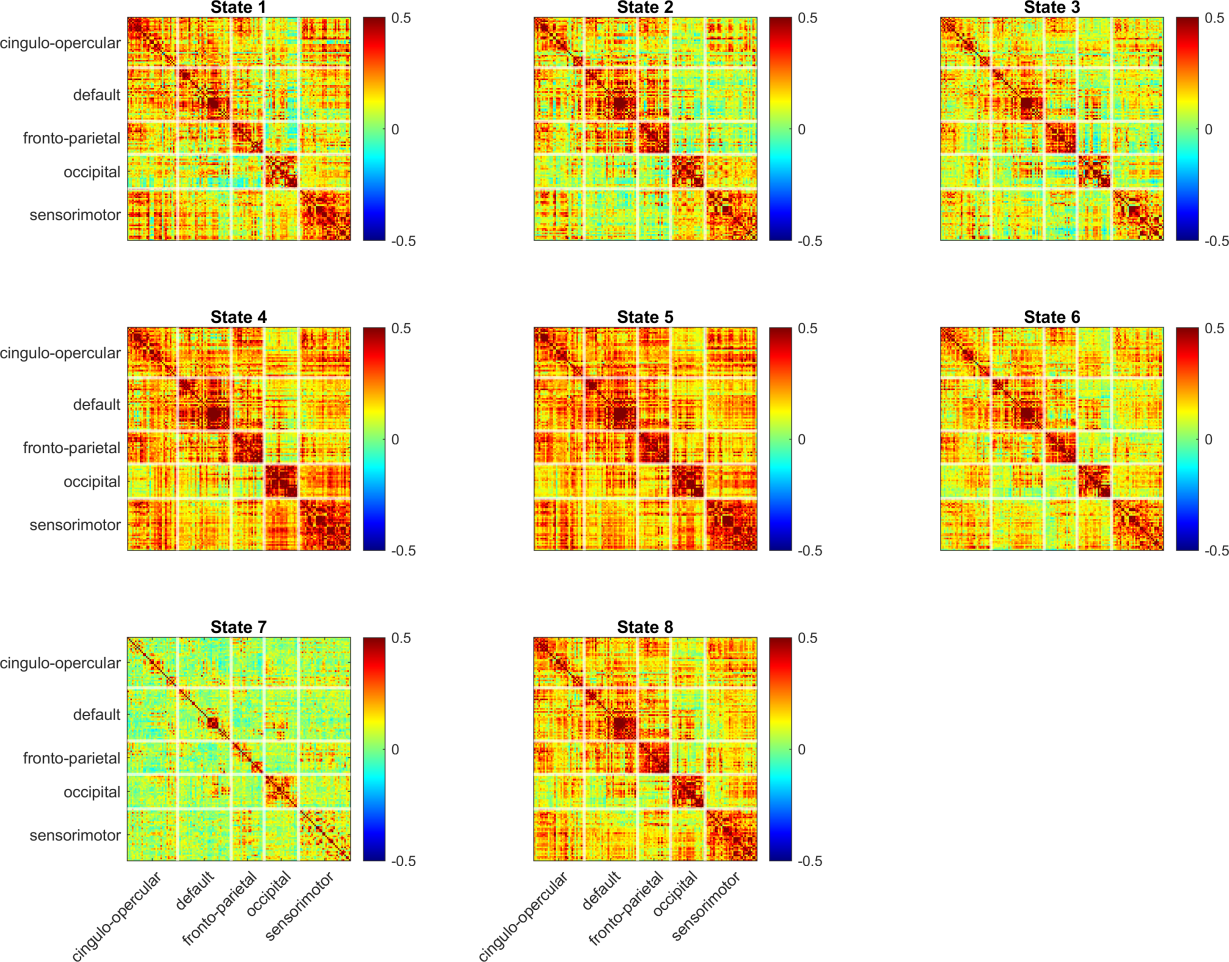
State-specific functional connectivity patterns identified by the hidden Markov model.

Based on the minimum free energy criterion, an HMM with eight recurrent spatial states was identified across all participants. Each state was characterized by a distinct pattern of within-network and between-network functional connectivity across the cingulo-opercular, default mode, fronto-parietal, sensorimotor, and occipital networks (**Figure 4**).

**Figure 5:**
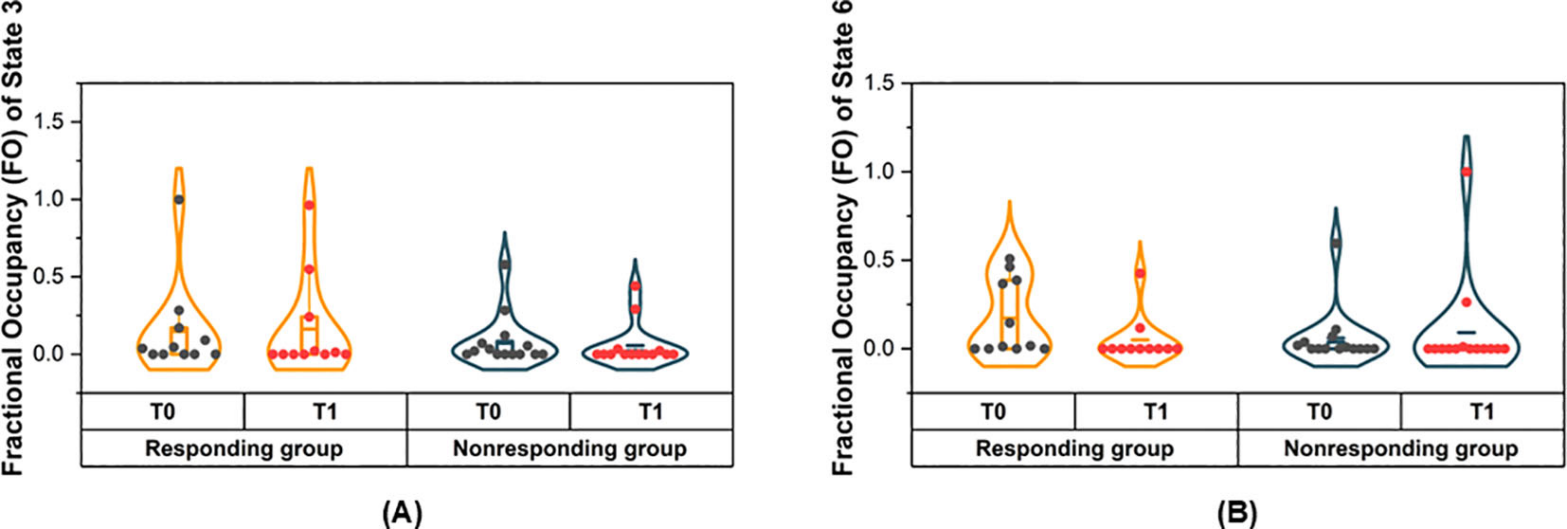
Fractional occupancy of HMM states before and after the intervention. Violin plots displaying the distribution of fractional occupancy (FO) for **(A)** state 3 and **(B)** state 6 at baseline (T0) and post-intervention (T1) in both the responding and nonresponding groups. The internal boxplots represent the mean values, with overlaid scatter points showing individual patient values. The orange outlines denote the responding group, while the dark blue outlines denote the nonresponding group. (FO, fractional occupancy; HMM, hidden Markov model; T0, baseline; T1, post-intervention).

Group comparisons of higher-order HMM-derived dynamic metrics showed that FO differed significantly between responders and non-responders for state 3 (95%CI 0.03(0.00–0.28), *P* = 0.033) and state 6 (95%CI 0.02(0.00–0.35), *P* = 0.038). Specifically, responders exhibited reduced FO in these states compared with non-responders (**Figure 5**). No significant between-group differences were observed in other dynamic measures, including SR and MDT.

### 3.5 Clinical correlations of significant neurophysiological indicators

**Figure 6:**
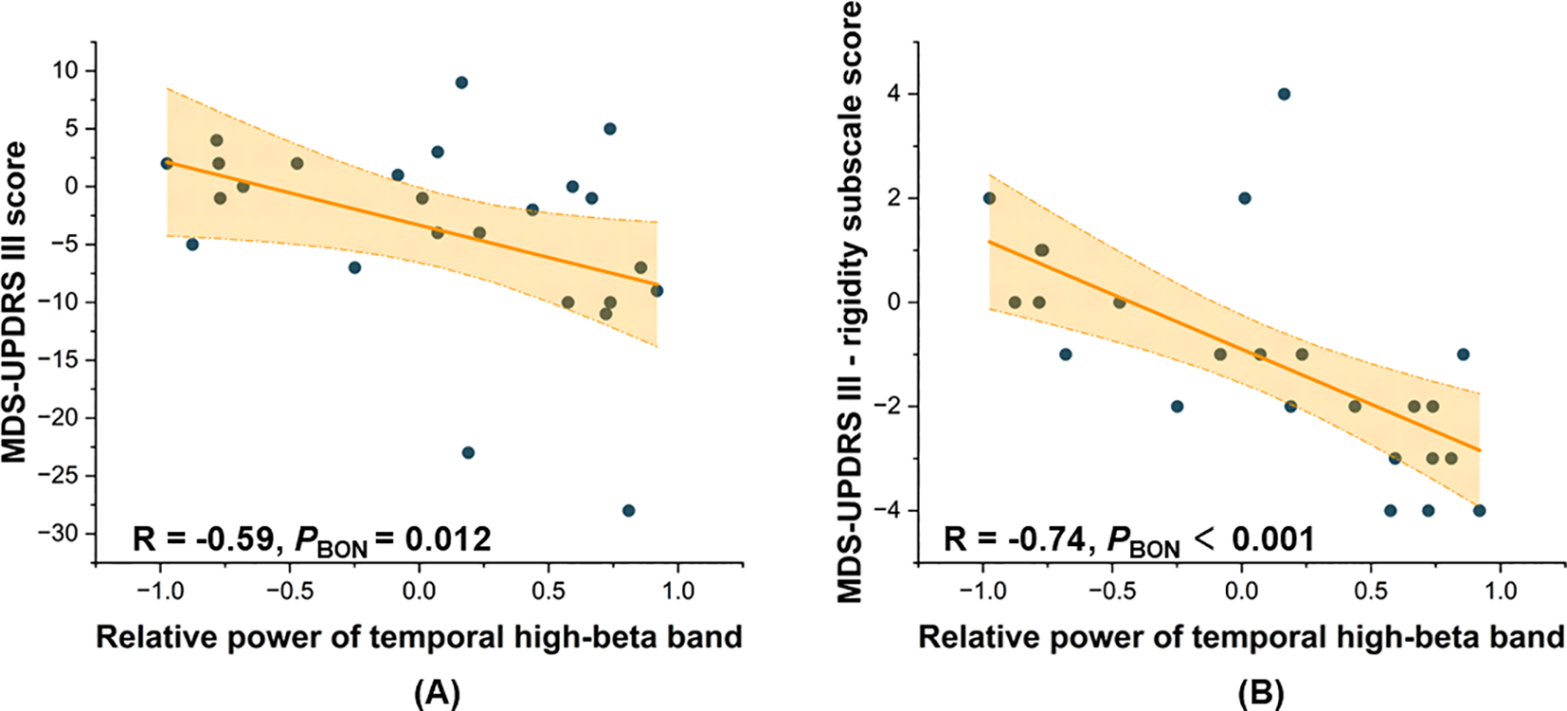
Clinical correlations of significant neurophysiological indicators. **(A)** Scatter plots display the clinical correlation of temporal high-beta (20–30 Hz) relative power with MDS-UPDRS III score differences. **(B)** Scatter plots display the clinical correlation of temporal high-beta (20–30 Hz) relative power with rigidity subscale score differences of MDS-UPDRS III.

Controlling for age and disease duration, partial correlation analyses were performed to examine the relationships between neurophysiological and neuroimaging markers that showed significant between-group differences and changes in motor symptoms. After Bonferroni correction, only changes in temporal high beta frequencies relative power were negatively correlated with changes in total UPDRS-III scores (R = -0.59, *P*_BON_ = 0.012) and rigidity subscores (R = -0.74, *P*_BON_ < 0.001) (**Figure 6**). No significant partial correlations were observed between other EEG or MRI-derived metrics and changes in bradykinesia, postural stability, or tremor subscores after Bonferroni correction.

## 4 Discussion

In this secondary analysis of an RCT, we examined multi-level neurophysiological features associated with motor improvement following Hi-tACS combined with MIRT in PwP. After the ten-day intervention, responders demonstrated clinically meaningful improvements in motor function. Compared with non-responders, these patients showed a convergent pattern of neural modulation, including increased temporal beta high oscillatory activity, reduced FC between the DMN and SMN, and decreased occupancy of diffuse, low-selectivity brain states. Together, these findings indicate that motor improvement following combined neuromodulation and rehabilitation is accompanied by coordinated changes across oscillatory, network, and dynamic brain organization levels.

A central finding of this study is the marked divergence between responders and non-responders in treatment-related changes of temporal high beta frequencies, which emerged as the only neurophysiological measure that remained significantly associated with the magnitude of motor improvement after correction for multiple comparisons. Converging evidence has established beta-band oscillations as a core component of PD pathophysiology (31). Compared with healthy individuals, PwP typically exhibit reduced cortical beta power, and this reduction has been linked to greater motor symptom severity (14). Importantly, the functional significance of beta activity appears to depend on both its cortical localization and its frequency-specific characteristics (32). Within the beta range, lower and higher frequencies have been proposed to reflect distinct pathophysiological mechanisms. While lower beta activity is more closely associated with dopaminergic depletion, higher beta frequencies are thought to support physiological motor circuit engagement and dynamic modulation during movement execution (33). Within this framework, the increase in temporal high beta frequencies observed in responders is unlikely to represent an exacerbation of pathological synchronisation. Rather, it may reflect a restoration or reorganization of physiologically relevant beta activity. This interpretation is consistent with prior evidence showing that pharmacological therapy, deep brain stimulation, and tACS can modulate aberrant oscillatory patterns and alleviate rigidity–bradykinesia symptoms (15, 34–37). Moreover, the temporal cortex plays an integral role in sensorimotor integration, proprioceptive processing, and higher-order motor planning, all of which are essential for effective muscle tone regulation and motor execution (38). Taken together, the ability to upregulate temporal high beta frequencies may index a preserved capacity for adaptive cortical reorganization, thereby constituting a neurophysiological substrate underlying interindividual variability in motor responsiveness to combined neuromodulatory and rehabilitative interventions.

Beyond regional oscillatory modulation, responders and non-responders also diverged at the level of large-scale functional network interactions. Specifically, responders exhibited a post-treatment reduction in FC between the DMN and the SMN, whereas non-responders showed an opposite pattern. This distinction is especially pertinent in PD, where extensive dopaminergic depletion and cortical α-synuclein pathology are recognized to disturb large-scale network organization and hinder the dynamic equilibrium between inwardly focused and task-related brain systems (39). The DMN represents inherent, self-referential cerebral activity and is generally inhibited during goal-oriented actions, facilitating the efficient functioning of task-relevant networks like the SMN (40–42). The SMN incorporates primary sensorimotor cortices and supplementary motor regions to facilitate motor preparation and execution (43, 44). Previous rs-fMRI studies have demonstrated abnormal connectivity within and between these networks in PD, suggesting a failure to appropriately segregate internally focused processing from motor control demands (18, 45). Excessive coupling between the DMN and SMN is thus regarded as an indicator of network-level inefficiency, potentially hindering motor function. The diminished DMN–SMN connection noted in responders may indicate a partial reinstatement of functional network segregation after the combined Hi-tACS and MIRT intervention. This balance may enhance sensorimotor processing by reducing distraction from internally focused cognitive activity during motor execution. In contrast, the heightened coupling observed in non-responders may signify a continuation of maladaptive network integration, aligning with compromised top-down motor regulation and less ability for network reconfiguration. Collectively, these findings suggest that individual differences in the ability to recalibrate large-scale functional network interactions may contribute to variability in motor responsiveness to combined Hi-tACS and MIRT in PD.

Beyond static FC, HMM enabled characterization of the temporal organization of large-scale brain dynamics by identifying eight recurrent connectivity states (19). These states exhibited heterogeneous patterns of within- and between-network interactions across the five networks. Notably, only state 3 and state 6 showed significant between-group differences in FO. Examination of their state-specific connectivity matrices indicated relatively distributed interaction patterns, with moderate coupling observed both within and across multiple networks and without clear dominance of any single functional system. Compared with more segregated states characterized by pronounced within-network connectivity, these configurations reflect reduced network specialization and greater cross-network integration. In PD, similar patterns of diffuse inter-network coupling—particularly involving DMN and SMN—have been associated with impaired dopaminergic modulation and reduced flexibility of large-scale brain dynamics (18). In the present study, responders demonstrated a post-intervention reduction in the fractional occupancy of state 3 and state 6, whereas non-responders showed persistence or relative increases in these states. This divergence suggests that motor improvement was accompanied by a shift in temporal brain dynamics away from distributed, weakly differentiated configurations toward more functionally specialized and efficiently organized network states. These HMM-derived differences should be interpreted as dynamic neural correlates of inter-individual variability in treatment responsiveness rather than direct indicators of causal mechanisms.

Collectively, these findings suggest that clinically significant motor enhancement resulting from the combination of Hi-tACS and MIRT cannot be ascribed to a singular neural mechanism. Responders demonstrated synchronized alterations across all levels of brain architecture, encompassing localized oscillatory activity, extensive network interactions, and dynamic brain state configurations. Significantly, whereas various neural characteristics distinguished responders from non-responders, only temporal beta high oscillatory changes were directly correlated with the magnitude of motor improvement. This pattern highlights the potential role of beta high oscillations as a proximal neural correlate of rigidity improvement, while network-level and dynamic changes may reflect broader contextual modulation that supports or constrains behavioral expression. Such a multi-level perspective may help explain the pronounced inter-individual variability observed in response to combined neuromodulation and rehabilitation interventions in PD.

Several limitations of the present study should be acknowledged. First, this secondary analysis exclusively investigated patients receiving combined Hi-tACS and MIRT, deliberately omitting the sham-control group from the parent RCT. This design choice was intentional: our previous study has already elucidated the neuroplastic mechanisms of MIRT alone, which primarily facilitates a shift in neural networks from sensory processing to higher-order cognitive control (4). Furthermore, because the parent trial demonstrated no significant group-level motor differences between the active and sham arms, the pressing analytical need was to understand the profound inter-individual variability within the combined treatment framework, rather than conflating the specific neurophysiological signatures with a sham-group comparison. However, a natural consequence of this restricted focus is that it does not allow for definitive causal inference regarding the independent effects of Hi-tACS compared to MIRT alone. While this design reflects common clinical practice in which neuromodulation is implemented as an adjunct rather than a standalone intervention, the specific additive effects of stimulation cannot be fully disentangled within the current analytical framework. Second, the relatively small sample size limits the statistical power and generalizability of the findings. However, this preliminary exploration provides important insights into the potential mechanisms underlying the combined intervention. Additionally, the relatively brief intervention period and the lack of long-term neuroimaging follow-up limit conclusions regarding the durability of the observed neural reorganization. Future studies with larger sample sizes, multi-arm designs to isolate independent and synergistic effects, extended longitudinal neuroimaging assessments, and predictive modelling approaches are warranted. Such designs may help determine whether baseline oscillatory or network-level features can prospectively identify individuals most likely to benefit from combined Hi-tACS and MIRT interventions, thereby supporting more personalized treatment strategies in PD.

## 5 Conclusions

In this secondary analysis of patients receiving combined Hi-tACS and MIRT, clinically meaningful motor improvement was characterized by coordinated alterations across oscillatory, network, and dynamic brain domains. Responders exhibited a pattern characterized by enhanced physiologically relevant beta-band activity, increased functional segregation between large-scale networks, and a shift away from diffuse, low-selectivity brain states toward more stable and differentiated dynamic configurations. Collectively, these findings indicate that individual differences in motor responsiveness to combined Hi-tACS and MIRT are associated with multiscale reorganization of brain dynamics, rather than isolated changes at a single neural level.

## Data Availability

The raw data supporting the conclusions of this article will be made available by the authors, without undue reservation.
